# A qualitative synthesis of practice-based learning from case studies on COVID community champion programmes in England, UK

**DOI:** 10.1186/s12889-023-17470-1

**Published:** 2024-01-02

**Authors:** Jane South, James Woodall, Jude Stansfield, Tom Mapplethorpe, Andrew Passey, Anne-Marie Bagnall

**Affiliations:** 1 School of Health, Centre for Health Promotion Research, Leeds Beckett University, Calverley Building, Portland Place, Leeds, LS1 3HE UK; 2 Corporate Strategy, Commissioning and Public Health, Kirklees Council, Huddersfield, HD1 9EL UK

**Keywords:** Pandemic, Volunteering, Community mobilisation, Health champions, Practice-based evidence, Case studies, Empowerment

## Abstract

**Background:**

Community-based volunteering supports outbreak management by extending reach into at-risk communities. This paper examines the application of a ‘community champions’ model in England, UK, during the COVID-19 pandemic. Evidence pre-pandemic shows that community champion interventions tap into social networks to strengthen connections with disadvantaged communities. During the pandemic, the UK government set up a COVID community champions funding award scheme for local authorities to develop local programmes that addressed emerging inequalities. The study aim was to identify transferable learning on community engagement in the pandemic by undertaking a secondary qualitative synthesis of practice-based case studies of local COVID community champion programmes.

**Methods:**

A systematic staged approach for synthesis of practice-based case studies was used. In total, 16 COVID community champion case studies, which were written by practitioners involved in local programme implementation and published by the Local Government Association, were included. Case studies covered aims, programme development and delivery, examples of activities and a discussion of learning. Framework qualitative analysis methods were used to code and organise data prior to cross case analysis. The final stage produced an overarching thematic framework that best represented descriptive and interpretive themes.

**Results:**

The results provide an overview of common features of COVID community champion programmes and emergent learning. All local programmes aimed to reduce health inequalities by involving at-risk communities in local prevention efforts, adapting the approach to local priorities. Two levels of community engagement were volunteer mobilisation and subsequent community-based outreach activities. Elements of capacity building, such as training and creation of networks, were common. The synthesis of practice-based learning found that stronger relationships with communities were regarded as a key mechanism to support more equitable prevention strategies. Other learning themes related to champion roles, community engagement strategies and programme implementation.

**Conclusions:**

By focusing on how community champion approaches were applied by local authorities in England during the COVID-19 pandemic, this study contributes to knowledge on volunteer mobilisation as a mechanism to improve public health communication and outreach. Notwithstanding the limitations of experiential evidence, the synthesis of practice-based learning highlights potentially transferable community engagement strategies for health protection and health improvement.

**Supplementary Information:**

The online version contains supplementary material available at 10.1186/s12889-023-17470-1.

## Background

Community engagement has been a core strategy in the global public health response to the COVID-19 pandemic, underpinning prevention efforts at population and community-levels [1, 2]. Community engagement interventions typically offer a means to reduce or mitigate health inequalities associated with the pandemic by working with groups whose socioeconomic circumstances place them at greater risk [3–5]. This includes the challenge of the ‘inverse equity hypothesis’ whereby more privileged groups are quicker to adopt new technologies, such as vaccination, leaving a ‘lag’ in rollout for the less privileged [6]. Meeting these challenges requires more than one-way communication of health messages. An alternative is to use robust community engagement strategies working in partnership with communities to reduce these inequalities [3, 5]. In this paper, we use a broad definition of community engagement as involving the active participation of communities, whether connected by geography or interest, in priority setting, and intervention design, delivery and evaluation [7]. This can include interventions focused on community empowerment, whereby communities lead action and eventually gain greater control over the conditions that affect health and wellbeing [7].

Past experience of outbreak management suggests that social mobilisation, which can be seen as a process of building community participation, often at scale, around a common purpose, and community empowerment are both key factors in promoting health and preventing disease during a health emergency and beyond [8, 9]. Community-based volunteering, through both formal roles and more informal contributions, has also been found to play a critical role in initial emergency efforts and subsequent support for affected communities [10, 11]. From a public health perspective, there is potential to link the contribution of volunteers to efforts to reduce health inequalities associated with the pandemic by working alongside underserved communities [5]. Findings from a rapid evidence synthesis on community engagement for COVID-19 prevention and control identified community leaders and individuals (volunteers) as some of the main actors who can contribute to ‘equity-informed’ responses to the pandemic through activities such as outreach and advocacy [12].

This paper looks at the application of a ‘community champions’ model in England, UK, based on the mobilisation of volunteers from disadvantaged and at-risk communities during the COVID-19 pandemic. Pre-pandemic in the UK, this model was an established public health approach [13, 14] endorsed by national community engagement guidance [15]. Community champions, also called health champions, are defined as active community members working to promote health and wellbeing or to improve conditions in their local community [15]. These are bridging roles [16] where volunteers act as connection points between public services and the wider community and faciliate comunication. The community champion approach tends to focus on informal volunteering and promoting health through natural conversations and social networks, although some training is usually given [14]. A rapid scoping review on community champion approaches for the pandemic response and recovery, conducted by the authors, found evidence that champions and similar volunteer roles were of value in improving connections in communicable disease prevention and for longer term health improvement programmes [17]. Globally, community champion approaches sit within a long tradition of community engagement interventions that recruit community members with credibility, empathy and access to social networks in order to engage with target communities on public health issues [18, 19].

In January 2021, the UK government introduced a COVID community champions scheme [20] to respond to the then emerging evidence of health disparities around COVID-19 prevalence, morbidity and mortality [21] including a disproportionate impact on ethnic minority communities [22]. An earlier paper presented to the government’s scientific advisory board had highlighted the potential for the community/health champion approach to be adapted to support NHS Test and Trace services, by enabling reach into particular communities and tackling low trust in government [23]. The first wave of funding allocated £23.75 m to 60 English local authorities to implement COVID community champion programmes [20]. A formula was used by the Ministry of Housing, Communities and Local Government, the department administering the scheme, to identify the 60 local authorities (out of a total of 298 in England) with populations disproportionately affected by COVID-19; at that time this included older people, ethnic minorities and people with disabilities [20]. The COVID community champions scheme provided targeted short term funding to rapidly improve and widen community engagement as part of local pandemic responses. Local authorities developed local plans to fit with the broad aims, scope and principles of the government scheme to reduce health disparities. However, they had flexibility to develop an approach, or a series of targeted activities, that worked for their local population and context. Some local authorities had already developed community champion schemes prior to receiving funding. The UK government reported that there were just over 4,600 champions recruited as part of the scheme by end of March 2021, who were credited with ‘playing a vital role in tackling misinformation and driving vaccine uptake’ [24]. In January 2022, a second round of funding allocated £22.5 m to a further 60 local authorities (including some who had received funding in the first wave) with a focus on supporting the vaccination programme [25]. The press release reported that over 14,000 community champions had been part of the scheme in 2021.

As local champion programmes became established, supported by the central government funding, this created opportunities for knowledge exchange and dissemination of learning from public health practice. Between 2021 and 2022, a series of practice-based case studies on local COVID community champion schemes were gathered by Public Health England (PHE) (later Office for Health Improvement & Disparities—OHID [26]) Healthy Communities team to support wider implementation. The case studies were then published by the Local Government Association (LGA) and made openly accessible on the LGA COVID-19 Learning Exchange, later on LGA Public Health Learning Exchange, as a way of rapidly sharing learning about local community champion programmes within local government and with the public health workforce [27].

This paper reports on a secondary qualitative synthesis of those COVID community champion case studies and draws out implications for public health practice. Practice-based case studies, also known as practice examples, are a recognised method for communicating learning from practice [28, 29] and may yield useful evidence for practitioners [30] and policy makers [31]. The COVID-19 community champion collection provided a set of rich narratives on how community champion approaches had developed in a period of change and challenge for public health. The primary aim of the study, which was led by an academic team in collaboration with the Office for Health Improvement and Disparities (OHID), was to identify, synthesise and disseminate practice-based learning from case studies of COVID community champions developed during the pandemic. A secondary aim was to highlight potentially transferable community-centred approaches to build resilient communities and reduce health inequalities.

## Methods

### Study design

Practice-based case studies are a form of experiential evidence typically presented in the form of a narrative or practice story about implementation in a specific context [32, 33]. Synthesis of multiple case studies can help identify key processes and factors influencing implementation across different settings or projects [34, 35]. Synthesis methods draw on qualitative research traditions for handling case study sources [34, 36]. For this study, we used a systematic staged approach for synthesis of practice-based case studies, originally developed for the What Works Centre for Wellbeing [37]. This approach provides a transparent and logical method in seven stages (Table 1) and has been adapted for other analyses of community engagement in the pandemic [38].

**Table 1:** A staged approach to the synthesis of practice-based case studies on COVID community champions

**Stages** [37]	**Community champions synthesis**
(i) Identify or develop a conceptual framework that helps define, categorise and select interventions of interest	Community champions are part of Public Health England’s family of community-centred approaches [13]. The rapid review, published by PHE in 2021 [17], identified types of champion approaches in UK public health practice
(ii) Identify websites and case study collections	The LGA COVID Community Champion collection comprised 13 case studies. A further three were identified through searching the LGA COVID-19 Learning Exchange (now Public Health Learning Exchange)
(iii) Search and select case studies that group round a topic or intervention approach	LGA COVID community champion case studies were written to a standard of reporting, containing common information fields. There was some variation as local approaches developed according to context and timing/stage of the pandemic
(iv) Organise the case study data using a template with common fields/domains	A structured data extraction template was used to organise and display the data extracted from practice-based case studies. Common domains included aims, setting, approach, participants, activities, outcomes, influencing factors and learning
(v) Use cross case analysis with matrices to develop the analysis and synthesis	Cross-case analysis was used to identify the patterns and differences between case studies, retaining the contextual information within individual cases. This stage used tables to summarise data, in line with framework analysis
(vi) Develop an overarching framework that explains the data and can be adapted as more case studies are analysed	An overarching thematic framework was produced that provided a good fit with the data. The emerging findings were checked and rechecked against the case studies in an iterative process to refine the analysis. The final framework was as presented in tables, with a conceptual figure representing key programme features
(vii) Write a narrative report of themes, with illustrative quotations alongside contextual information	The final stage was a narrative account of results and themes with quotations and/or practical examples

### Data sources

The COVID community champion case studies were originally collated by OHID staff (national and regional teams) to support wider implementation in public health practice. Three of the authors were involved in this process as part of their previous roles with OHID (TM, JAS, JS). An invitation to provide a case study went out to all local authorities receiving funding through the government scheme in March 2021. Regional OHID teams also helped identify local authorities willing to share their learning. Some local leads had already presented at learning webinars held by the government scheme. No selection criteria were applied by OHID staff at this stage as dissemination of learning from public health practice was encouraged whatever model was used in local implementation.

Local authorities who agreed to provide a case study were asked to document their learning by completing a common template based on PHE’s (later UK Health Security Agency) practice example collection [39]. The practice example collection uses a common process for collection, review and curation of practice-based case studies [29]. This process was simplified for the COVID case studies to ensure that perspectives from practice were captured in a timely fashion, given the pressures on local public health teams during this period. The template covered key content including programme aims, population groups, delivery arrangements, reported outcomes and learning (Table 2). Following a process of review by OHID and LGA staff to ensure consistency of presentation, eleven case studies were published in the public domain on the LGA COVID Learning Exchange (now LGA Public Health Learning Exchange [27]) between June 2021 and February 2022, and a further two in October 2022.

**Table 2:** Case study template fields

Title of scheme
Local area(s) covered
How does this scheme support your local COVID-19 response? Are there other priorities for your champions?
How did the scheme come about? When did it first come about?
Please briefly describe your local population. Does your scheme target any specific population groups?
How does the scheme work? Which organisation or groups are involved?
How are champions recruited?
How are champions trained and supported?
How do you engage and communicate with champions?
Has the scheme been evaluated in any way?
What outcomes has the scheme led to?
What has been your key learning from the scheme to date?
How are you planning to develop your scheme moving forward?
Contact details for further information

### Sample

The sample for the synthesis was all COVID champion case studies on the LGA COVID-19 Learning Exchange that offered an account of how local champions programmes worked in practice and what learning emerged. Of the 16 case studies included in the synthesis, 12 received government funding in the 1st round (January 2021). A further 3 received funding in the 2nd round, although the case studies reported earlier activity. One case study area did not receive any government funding and the champions programme was initiated in the community. Twelve case studies had been collated by the OHID Healthy Communities team (TM, JAS) using the common template [c1-c12]. No selection criteria were applied as the call to submit a case study had been open to all local authorities that were part of the government champions scheme. A further case study presented a strategy with four shorter cases written by community-based organisations [c13]. Three additional case studies were identified through searching the LGA COVID-19 Learning Exchange using the term ‘community champion’ in August 2022 [c14-16]. One was a structured case study similar in length and content to the other case studies and the other two provided shorter accounts of learning.

### Analysis

We used framework analysis methods to guide the qualitative analysis [40], starting with coding, organisation of data and development of initial thematic charts. Each case study, which typically comprised 8–10 pages (range 4–12 pages), was read and reread to gain familiarity. A structured coding framework was developed to code the case study reports as textual data. Four thematic charts were developed: 1) programme development and delivery; 2) champion roles; 3) community engagement; 4) learning and outcomes. At this stage, coding categories mirrored headings of the template (Table 2). Some fields were further broken down; for example, recruitment was split into three codes: recruitment methods, description of champions recruited, numbers recruited. In line with a framework analysis approach [40], coded data were then summarised and charted on an Excel sheet, with each case study as a row and thematic categories as columns. Some text extracts were included in these charts.

The next stage was a cross case analysis to identify the patterns and differences between case studies, retaining the contextual information within individual cases [34, 36]. This stage involved refining the thematic charts to summarise and organise the data. A further round of analysis was undertaken to identify a set of themes best representing reported learning points and additional reflections on programme development. Brief vignettes summarising local approaches were also developed to help retain contextual information about local programmes.

### Synthesis and reporting

The final stage involved developing an overarching thematic framework that provided a good fit with the data and best represented descriptive themes (about programme features) and interpretive themes (about learning). A figure was used to display emerging relationships between public health/services; community champions and communities and learning was grouped into four major themes (reported below).

To ensure rigour of the qualitative analysis process, the steps and initial coding framework were agreed by three researchers (JS, JW, JAS). After initial coding and development of thematic charts by a single researcher (JS), another researcher (JW), who was independent from OHID, undertook an audit of the quality, consistency and completeness of the analysis by comparing a random sample of case studies (*n* = 6) with thematic charts, line by line. The remainder of the case studies (*n* = 10) were then read by other members of the academic team (AP, JAS, AB) and the thematic charts checked. Cross case analysis was discussed by all the team and the final narrative was agreed by all authors.

### Ethics

The synthesis used case studies that were already published in the public domain as part of the agreed work programme of OHID Healthy Communities team. Local leads completing case studies did so in response to an open invitation and agreed the final version, understanding that the case study would be made publicly available.

The synthesis study had ethical approval through Leeds Beckett University and was agreed by OHID and the LGA. The results represent a synthesis of programme implementation and learning during the pandemic and therefore specific case studies, areas or organisations are not identified.

## Results

The synthesis provided insights into local COVID community champion programmes, which were developed in a relatively short time period to address health inequalities associated with the pandemic. The set of 16 case studies covered a diverse range of local authorities, both upper tier authorities and district councils. Table 3 presents a summary of the characteristics and programme features of the case studies included in the synthesis. Additional File 1 provides publication details of the included case studies, with access and publication dates.

**Table 3:** Summary of community champion programmes and roles

Case	Date published	Programme approach	Target population groups	Settings	Main champion roles	Reported numbers recruited
c1	10/12/2021	Community champion programme was a major component of partnership work to increase the reach and effectiveness of COVID communications. Delivery through VCS organisations who recruited, trained and supported champions	Ethnic minority communities Migrants, asylum seekers & refugees People with a disability	Community settings (including faith) Workplaces/ local businesses	Outreach activities Awareness raising & communication of public health messages Signposting to information/support Building trust	Not reported
c2	15/09/2021	Creation of network of community partners (grassroots organisations and faith groups), coordinated by Race Equality Network. Lead champions recruited other champions and facilitated 2-way information flows with communities	Ethnic minority communities People with a disability	Community settings (including faith)	Outreach activities Awareness raising & communication of public health messages Signposting to information Developing/tailoring communication materials Community leadership Feedback of community insights	300 including 49 lead champions
c3	17/01/2022	Community-initiated programme, later rolled out across district. Focused on creation of ‘network of informed and aware citizens’ who raised awareness and understanding of vaccination and managing COVID risk	Ethnic minority communities Young people People with a disability	Community settings Workplaces/local businesses	Awareness raising & communication of public health messages Promotion and practical support to vaccine clinics and community testing	200
c4	31/10/2022	Pre-existing community connector programme adapted to the pandemic response. Community connectors were employed by VCS organisations to connect with communities and help design culturally sensitive services. Approach based on asset-based principles using appreciative inquiry as basis for community conversations	Ethnic minority communities Older people People with a disability Older people Other vulnerable groups	Community settings	Outreach activities Awareness raising & communication of public health messages Signposting to information/support Promoting health & wellbeing Strengthening social connections Community leadership	10 community connectors
c5	13/12/2021	Population-wide approach focused on capacity building in workplace and community settings. Pre-existing MECC scheme extended to other vulnerable groups and settings. Training supported champions to cascade information to communities	Ethnic minority communities Migrants, asylum seekers, refugees Other vulnerable groups eg. carers, veterans	Community settings Workplaces/local businesses Local services Schools	Awareness raising & communication of public health messages Promoting health & wellbeing Signposting to information/support Engaging in training/support for local services and community groups	Over 500 Also 6000 + junior champions
c6	05/08/2021	Strengthening local infrastructure and collaboration with VCS organisations to improve outreach, communication and volunteering. Champions (people or organisations) worked within community action network	Ethnic minority communities Young people Older people People with a disability Homeless/rough sleepers	Not specified – across district	Awareness raising & communication of public health messages	98 (across 55 local organisations)
c7	10/08/2021	Pre-existing health champions programme based on neighbourhood work, community and personal development. Additional COVID health champions role to support pandemic response. Programme gathered community insight to shape services and address inequalities	Other vulnerable groups	Community settings	Awareness raising & communication of public health messages Promotion and practical support to vaccine clinics Signposting to information/support Promoting health & wellbeing Direct support to food banks, shopping services Strengthening social connections Community leadership Feedback of community insights	195
c8	31/10/2022	Community champion programme focus on improving communication and vaccine uptake. Grants given to VCS organisations and groups to reach target communities, recruit champions and build capacity for engagement activities. A learning & support network facilitated communication and sharing community insight	Ethnic minority communities People with a disability Other vulnerable groups eg carers	Community settings Local services Workplaces/ local businesses	Outreach activities Awareness raising and communication of public health messages Promotion and practical support to vaccine clinics Feedback of community insights	80
c9	14/06/2021	Establishment of a health equity collaboration to improve messaging and communication to at-risk populations. Supported 2-way information flow with communities. Range of volunteer roles including community influencers using mass media and COVID chat volunteers	Ethnic minority communities Migrants, asylum seekers & refugees People with a disability	Not specified – across district Workplaces/ local businesses	Awareness raising & communication of public health messages Community leadership Feedback of community insights	Not reported
c10	17/10/2021	COVID champions network was key mechanism supporting 2-way information flows with communities. Grew from network of funded VCS organisations to include staff, residents and community leaders. Public health facilitated network and provided grants	Ethnic minority communities Older people Other vulnerable groups	Community settings (including faith)	Awareness raising & communication of public health messages Promotion and practical support to vaccine clinics Organising community settings for vaccination and community testing Feedback of community insights	60 Also 244 in COVID champions network
c11	04/10/2021	Comprehensive community champions programme built on pre-existing whole system communities strategy. Communication and gathering community insights occurred through local networks and COVID community engagement groups. Champions discussed wider health determinants as well as pandemic issues	Ethnic minority communities People with a disability Other vulnerable groups	Community settings	Outreach activities Promoting health and wellbeing in communities Strengthening social connections Feedback of community insights	Not reported
c12	19/08/2021	Programme focused on community support within pandemic. Volunteering service based on ‘wellbeing friends’ offering 1:1 telephone support to increase wellbeing and reduce social isolation of vulnerable adults. Also a wider champions network to improve messaging in communities	Other vulnerable groups	Not specified	Befriending and telephone-based support Social support and reduction of isolation Awareness raising & communication of public health messages	74 wellbeing friends 650 in network of community champions
c13	10/12/2021	Four case studies developed by VCS organisations; 3 representing specific ethnic minority populations and 1 working with young people from diverse ethnic minority communities. VCS groups received grants, mobilised staff, volunteers and community leaders and led design and delivery of engagement activities through community networks	Ethnic minority communities Migrants, asylum seekers & refugees Young people People with a disability	Community settings (including faith)	Outreach activities Awareness raising & communication of public health messages Social support and reduction of isolation Feedback of community insights	Not reported
c14	03/02/2021	Programme developed early in pandemic initially to support COVID messaging, later used for vaccine roll out. Champions as resource in public health system because they tackled barriers and built trust	Not reported	Not specified	Awareness raising & communication of public health messages	585
c15	24/02/2022	Programme built a network of champions and local grassroots organisations. Mix of supporting local health services and community-led outreach activities. Some activities supported by small grants. Emphasis on community empowerment and working with trusted messengers with cultural competence and language skills	Ethnic minority communities Young people Other vulnerable groups	Community settings	Awareness raising and communication of public health messages Outreach activities Awareness raising and communication of public health messages Signposting to information/support Promotion and practical support to vaccine clinics Developing/tailoring communication materials Leading and designing community activities	75
c16	03/02/2021	Community champions integral to partnership approach across various levels of local government. Emphasis on champions as trusted messengers who communicated messages, tackled disinformation and translated	Not reported	Not specified	Outreach activities Awareness raising & communication of public health messages Translation/tailoring of communication materials	30

**Fig. 1:**
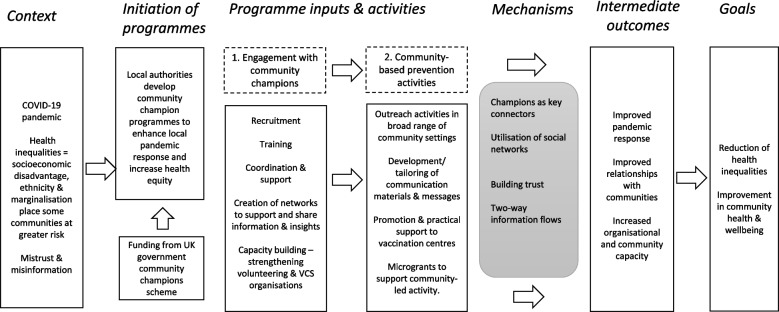
Programme logic for COVID community champions

Synthesis results are presented in two sections. The first section provides an overview of the features of local programmes, highlighting similarities and differences in approaches. Figure 1 presents the programme logic of COVID community champion programmes and roles, based on our case study synthesis findings. The second section reports on the four primary themes summarising practice-based learning around the utilisation of community champion approaches as part of local pandemic responses.

### COVID community champion programmes—key features

The case studies provided detailed accounts of local programme development and delivery. Although each local authority developed their own bespoke approach dependent on context and community need, there were also common elements. We report now on the scope and aims of community champion programmes and elements of implementation such as training. Results are distilled into a simple logic model summarising how programmes worked and the link between context, community engagement activities, mechanisms and outcomes (see Fig. 1).

Broadly, local programmes aimed to reduce health inequalities and to improve the pandemic response by working with at-risk communities (Table 3). This could be through improving communication, increasing knowledge and awareness of COVID, promoting risk reduction behaviours, increasing vaccine uptake or gaining understanding of the challenges faced by communities. Some programme aims were articulated in terms of capacity building and improving relationships with communities in order to address inequalities and exclusion. Eight case studies reported supplementary aims to improve health and wellbeing outcomes as part of long term health strategies [c1, c4-8, c11-12] and one had a focus on the reduction of social isolation associated with the pandemic [c12].

In terms of target communities, programmes reflected the purpose of the government scheme to work with groups disproportionately affected by COVID-19 (Table 3). Most case studies made reference to health inequalities and vulnerable populations, with six emphasising high levels of socioeconomic deprivation in their area and a further six highlighting ethnicity as a determinant of health. Local strategies often identified specific underserved communities, for example migrant communities. One case study explicitly recognised the issue of intersectionality across different vulnerable groups [c9].

The government scheme allowed for local adaption as each local authority developed their own bespoke community champion programme to address health need. Where reported, initial design tended to be led by public health and local authority staff, whereas the development of champion activities was mostly done in partnership with Voluntary and Community Sector (VCS) organisations and other local partners, such as health services. Results confirmed a shared rationale that community champion roles were seen as a means to improve outreach and engagement with underserved communities. There was a broad distinction between programmes that focused on building a cohort of community champions [eg c2, c7, c15], in some cases building on pre-pandemic schemes, and those programmes that gave emphasis to building integrated partnerships, where champions were one strand of community engagement activity [eg c1, c9, c11] (see Table 3). A cross cutting theme was the creation of participation structures where public health and other staff could engage directly with community champions, opening up opportunities for shared decision making. A minority of programmes adopted an explicit empowerment approach aiming to increase community leadership [c7, c11, c13, c15].

Elements of capacity building, such as training, support, creation of networks and investment in VCS organisations, were seen across all case studies. These components formed an infrastructure that supported the development of a cohort of community champions able to take on roles within the local pandemic response. Multiple recruitment methods were used including direct recruitment of members of the public to become champions, typically through open calls posted online, in print or via social media, and recruitment through trusted organisations, who were able to tap into their volunteer base and social networks to extend volunteering opportunities. Some case studies reported recruitment of workplace champions through local businesses and services. The motivations of community members to take up a champion role were not reported.

Brief training to prepare for the role was a common feature of COVID community champion programmes, although training varied in scope and intensity. Training was mostly delivered online, due to social restrictions. Some programmes provided a core training package, with typical content covering information about COVID-19 and prevention, combined with communication skills. Additional topics included standards of conduct, tackling misinformation and social media skills. Two local authorities, both of which had existing champion schemes, offered accreditation for course completion [c5, c7]. Being a champion is normally a volunteer role, but there were two exceptions to this. One programme employed a small cohort of community connectors [c4] and another offered some payment for participation in training (and to support ongoing engagement, such as mobile phone costs) [c2].

Results highlighted programmes working at two levels of community engagement; first, engagement with a cohort of community champions and second, community-based prevention activities involving champions engaging with community members (see Fig. 1). To support programme delivery and coordination, community champions were mostly hosted in VCS organisations. Ongoing support was generally provided by those host organisations and sometimes involved volunteer managers or nominated champions who were able to cascade information. A common feature was the establishment of communication channels, such as email briefings and online meetings, that allowed for dialogue with champions as the pandemic response evolved. Some case studies described the formation of wider champion networks that provided coordination, peer support and opportunities for knowledge exchange. Networks were a place where champions, or organisations coordinating champions, would be updated, share good practice and raise any issues that they were encountering. Meetings could be a space for dialogue and interaction with health professionals and local authority teams.

All case studies reported outreach activities involving champions working directly with community members. Activities took place across a diverse range of settings including faith settings, schools, shops and community spaces, such as parks. In general, there was a mix of neighbourhood-based activity, including support for vaccine clinics, combined with outreach to specific communities through VCS and faith-based organisations. Due to social restrictions associated with the pandemic, online outreach through social media was also reported. Two case studies described workstreams working with schools and young champions [c5,c7]. Three described the use of microgrants as a key strategy to build community-led activity around prevention [c3, c13,c15]. While case studies reported on the role of champions in supporting local pandemic responses, community engagement was generally founded on building improved relationships between local authorities and communities and not on using champions simply as a resource for activities.

### Learning from COVID community champion programmes

The case studies reported practice-based learning from implementing local COVID community champions programmes. Through the synthesis, we identified four major themes on learning:

- Stronger relationships with communities
- Champions as key connectors
- Effective strategies for community engagement
- Programme implementation.

These themes are interconnected as roles and relationships thread through different aspects of implementation. The first two themes are relational and discuss how trust and communication grew in local systems due to more intensive community engagement and specifically because of the role of community champions as trusted community members with social connections. The third and fourth themes summarise the practical learning from implementation and what engagement activities worked to overcome communication barriers. Figure 1 summarises how roles, relationships, and community engagement activities are connected.

### Stronger relationships with communities

The emergence of stronger relationships with communities was an overarching theme and the primary mechanism for improving the pandemic response. Building trust between communities and public authorities was found to be a key facilitator at all levels in local systems. Case studies typically related how trust was built up slowly from situations where there were low levels of trust and misinformation was circulating:

“Trusted relationships with key people across the local system enables true partnership working and enables sharing of intel and insight from communities. Sharing power and responsibility and learning together has helped to build rapport and respect.” [c5]

A major theme was the growth of communication channels linking public health teams with community champions and VCS organisations. This facilitated bilateral flows of information from and to communities on issues such as vaccine hesitancy and adopting COVID-safe behaviours. Engagement structures, such as networks and online forums, often allowed immediate and direct flow of intelligence between the different parties:

“[…]we cannot expect our champions to simply distribute our messages, adopt our approaches and signpost on our behalf without ensuring we are there to respond to their issues and queries – it must be a two-way relationship with open lines of communication.” [c10]

Case studies described approaches evolving opportunistically where relationships improved, trust was built, and the local response evolved. Some programmes developed a layered approach, working through grassroots VCS organisations embedded in specific communities, which in turn gave access to lived experience in the pandemic. The benefits of two-way communication processes were articulated across case studies. Integration of community insight into the pandemic response enabled more culturally appropriate communication and barriers preventing uptake of services to be addressed:

“The programme helped to ensure that vaccination centres were more culturally sensitive of the needs of specific groups including Black and minority ethnic communities, sex and gender minorities; it helped building confidence in local and national authorities around the management of COVID, including trust in the vaccine and services administering it.” [c4]

Several case studies emphasised how two-way communication had improved their strategic decision making around the pandemic response:

“Across the broad range of activity, two-way open dialogue was encouraged and facilitated to provide insights into community issues and concerns, to feed into and influence strategic decision making to inform future activity and approaches.” [c15]

### Champions as key connectors

The value of champion roles as a means to connect better with target communities was a major theme. Across the case studies, there was a common understanding of community champions promoting health in the places where they lived and worked and emphasis was given to their ability to reach people who were disengaged, isolated or underserved. Community champions were key touch points in communities, able to convey information and feed back concerns:

“It has been invaluable during this pandemic to have key individuals within local communities, whose role it is to communicate information clearly and consistently, and feedback queries, concerns and insights. Aside from the obvious practical support provided by champions in terms of testing and vaccination, having COVID-aware ‘eyes and ears’ in local communities has been really helpful in terms of adapting comms messages and targeting local campaigns, as well as in assessing the public mood more generally.” [c3]

Peer-to-peer communication and use of social networks were regarded as primary routes to connect with individuals and communities where misinformation and mistrust might be present. For some programmes, the emphasis was on champions within grassroots community-based organisations, as this was seen to increase reach into underserved communities. One community organisation explained:

“They (champions) also combated disagreement and criticism in a unique way choosing not to dismiss the feelings and emotions, but inviting the conversation and creating a dialogue. This proved to be an effective way of helping to remove concerns and change attitudes.” [c13].

Given the focus of the government funding, many case studies reported on the critical role of champions in supporting COVID vaccination roll out. This involved promotion of the vaccine, actively addressing misinformation, and in some communities, supporting vaccine delivery. An example was given of champions addressing transport barriers by creating a local taxi scheme to take people to the vaccine clinics [c10]. Another case study reported that champions had “provided accurate information, community reassurance and signposting” to support the roving vaccination bus, women only vaccination clinics and enhanced testing work [c15].

### Effective community engagement strategies

The case studies provided rich accounts of successful community engagement. Learning emphasised the importance of establishing effective communication pathways and champions as trusted messengers. There was an interplay between methods used by organisations (both public services and VCS organisations) and methods used by champions, with considerable overlap where communication tools were jointly designed. The breadth of communication methods and tools included:

- Social media (eg WhatsApp groups, Facebook, Twitter)
- Web-based communication eg. online forums, podcasts and webinars
- Videos and TikTok content with key messages from community champions and local leaders
- Community meetings and local events
- Peer-to-peer work with individuals and groups
- Community radio
- Posters, leaflets, banners and T-shirts

Sociocultural barriers linked to inequalities in access to services were noted in several case studies. These included language barriers, low literacy and experience of discrimination. A major theme was the importance of tailored information to meet the needs of diverse communities. Involving local leaders, including faith leaders and local clinicians, could help give credibility to messages. Translation to other languages was also a cross cutting theme. Almost all case studies described how champions had agency in contextualising public health guidance, drawing on their local knowledge:

“We do not micro-manage their work in this regard, trusting that they know their contacts and communities (incl. what they are most concerned or confused about) and will share what is of most value.” [c10]

“Each community delivers core messages in different ways, relevant to the best mediums and approach identified for those communities.” [c9]

Tackling misinformation by building trust, openness and respect was a key theme. Misinformation spreading within communities and via social media was identified as a significant barrier, which could hinder community engagement and uptake of vaccines. Regular communication and sharing community insights in partnership groups and champion networks helped develop public health strategies and effective messaging.

“It really is two-way feedback. We talk about what has been happening and they provide feedback about the messages they hear at community level. It really helps us understand how messages are landing and where the concerns are.” [c16]

An alternative approach was encouraging the participation of champions, and grassroots organisations, in the co-design of local activities and communication resources, such as videos and posters [c2, c5, c11, c13, c15].

### Programme implementation

There was a strong emphasis on the learning through designing and delivering COVID community champion programmes, in a constantly changing context. Local data (predominately drawn from national public health data sets available at local level) combined with community insights helped identify programme priorities and develop tailored solutions. Local adaptation and flexibility were seen to be key:

“What works in one local area or with one demographic may not translate directly to others. Have a plan and look at other programmes for ideas but, ultimately, let your programme be driven by your capacity and the needs of your target audience(s), not just by projected outcomes.” [c1]

The significance of adopting an open, non-hierarchical approach to partnership working, including with community-based organisations and communities was a cross cutting theme linked to addressing health inequalities:

“The importance of engaging and working collaboratively with partners at a grass-root level. These partners know their local communities best.” [c2]

Creation of community champion networks opened up a shared space for knowledge exchange and collaboration:

“Network members meet on a monthly (originally fortnightly) basis. These meetings allow champions to update each other (and us) on key pieces of work that they are involved in, to raise any queries or clarifications and to ask for advice or ideas from colleagues. We ask that they share anything they have produced that may be of value to other champions (e.g. comms / social media assets, videos, imagery, resources, etc.) and the network chair e-mails out any information received in this light between meetings.”[c10].

Funding was identified as a major facilitator both at programme and community-level. Some case studies explained how short term government funding had been critical to developing a scaled response and outreach. For a minority of areas with existing champion or similar health improvement schemes, these could be extended to new settings and communities:

“Having an established MECC [Make Every Contact Count] programme in this way was an enabling factor in terms of developing and standing up this aspect of our local response so quickly and effectively” [c5]

Overall, investment in VCS organisations to enable recruitment of champions and community engagement activities was a route to building community capacity. Where programmes used micro grants, typically given to support community-led activity, this was perceived to be a key facilitator that supported outreach.

Most case studies described how local programmes had undergone a series of cycles of development in responding to the challenges of the pandemic. There was little discussion of what had not worked, partly as this was not a requirement for reporting. Aspects that were identified as challenging included tensions between promoting government guidance and addressing local needs. Changing national guidance increased the time and effort needed to communicate new messages and more generally, made community engagement harder. Other challenges included achieving a balance between maintaining regular communication between public health professionals and champions and allowing for champions to have agency in spreading messages.

## Discussion

Community champions is a community engagement approach involving community-based volunteers who provide the connections to effect better relationships between health services and disadvantaged communities [15, 17]. This approach was adopted in England, UK, due to the potential of champions to reach into and engage communities disproportionately affected by the pandemic [23]. The synthesis of learning presented in this paper shows that COVID community champions was a relevant and highly adaptable intervention to support community-based prevention efforts during the pandemic. As results indicate, champion programmes were set up rapidly and quickly evolved according to local need. Through the cross case synthesis, we were able to identify core components of COVID community champion programmes, framed in a logic model (Fig. 1). In terms of influencing factors, the scope of the government scheme, which was focused on the champion model as a potential route to reduce disparities, will have influenced implementation; however, local authorities were given considerable flexibility to develop local approaches. Our results confirm that local context was important and informed delivery. Activities were often shaped by earlier engagement work with communities, including where existing community champion programmes were in place.

Broadly, this insight into public health practice in a challenging context supports a wider evidence base on similar roles in communicable disease control and in promotion of health and wellbeing [12, 17]. In particular, the COVID community champion approach shares some similarities with the Popular Opinion Leader model, which has a focus on individuals from at-risk groups able to influence others through their social networks [5, 41, 42]. An earlier rapid review in 2021 noted that little robust evidence on COVID community champions had yet emerged [17]; however, there were some reports of similar approaches being adapted as part of the pandemic response in other countries [5, 43, 44]. From the perspective of UK public health practice, this approach appears most useful when working with marginalised communities who have the greatest health risks and face significant barriers to receiving health messages and accessing prevention services.

Community mobilisation in the pandemic was significant in the UK [11] and internationally [45, 46]. This study adds to understanding of how volunteering models were applied in developing local pandemic responses. The synthesis identified recruitment strategies and the need for multiple methods; but there was no information in the case studies on the motivation of those volunteering for the role of community champion. This could be an important area for future research as there are likely to be a range of motivations [11], which may differ from the priorities of national and local government. Themes on the value of informal volunteering in the settings where people live, work and socialise echoes findings of a review by Whittaker et al. on informal volunteerism in disasters and emergencies [10]. The authors recommend an approach to volunteering that seeks to recognise and harness existing community capacities and assets [10]. Further evaluation of the government champions scheme is warranted however, funding appeared to be an accelerant for local programmes and was used to build community capacity and volunteering in many areas. Looking forward, there are risks to the longevity of community champion programmes set up in response to short term government funding. Lack of sustainable funding may also impact those VCS organisations working with communities experiencing socioeconomic disadvantage.

The case studies provided many examples of communication of health messages through natural social networks. Acknowledgement of the value of community knowledge is a critical part of community-centred approaches in public health [47]. For COVID community champions, a strong finding was the facilitation of two-way communication between communities and public health teams. This suggests that the champion approach is not a transactional model, but a relational one where champions become critical connectors. The learning about effective community engagement based on strong relationships with communities and bilateral information flows echoes lessons from the Ebola emergency [8, 9]. The finding that building trust between communities and public authorities was a key facilitator in local public health systems [48] supports evidence from the COVID-19 National Preparedness Collaborators that trust was a key factor explaining variations in infection rates between countries [49].

### Application to practice

This study contributes to practice-based learning on community engagement in the pandemic. Learning from implementation emphasises the flexibility of a champion approach. This is not a standardised model, yet despite the inevitable variation between and within case study areas, there was consistency with key elements of the champion model identified in the rapid review [17]. Common programme features (see Fig. 1) are:

- recruitment from target communities with high health needs
- training to give people confidence to take on a champion role in their community
- light touch support and supervision, with regular communication
- an open, inclusive approach to partnership working with local organisations, including VCS organisations
- creation of opportunities for champions to shape activities and services.

These programme features are potentially transferable and could be used to guide the establishment of new champion programmes either in emergencies or in the context of addressing longer term health inequalities. The programme logic presented in Fig. 1 could be adapted as a framework for planning and developing community champion schemes.

In terms of what works for community engagement and volunteer mobilisation, our findings indicate that implementation should be considered at two levels (i) recruiting and training a cohort of champions, linked to capacity building and (ii) the subsequent development of community-based prevention activities led by champions, including outreach and support to vaccination services (Fig. 1). There is a risk that community engagement in an emergency, such as the pandemic, becomes driven by top-down priorities decided by national and local government. The COVID community champions case studies showed that this was rarely the case as stronger relationships and bilateral flows of information were valued outcomes in most case studies. The implications are that approaches combining top-down and bottom-up elements to develop long-term trusted relationships between public health and communities is warranted. Similar learning also emerged from Ebola outbreak [8, 9].

Policy makers and practitioners should consider the benefits and risks of short term targeted funding to build community engagement activities rapidly in emergency situations. Issues of sustainability supported by longer term investment in volunteering and community infrastructure, such as champion networks, also need to be considered as part of community-level action to reduce health inequalities, build emergency preparedness and community resilience. Transferability of the community champion model would need further testing in other contexts but achieving health equity though better community engagement is a shared public health challenge. There are some similarities with other community-based prevention approaches used in the pandemic outside of the UK [5, 43, 44], opening up possibilities for greater sharing of learning on community mobilisation within public health.

### Limitations

While practice based case studies are a recognised source of learning about public health in the field [28, 35], data are not collected in a robust and systematic way to allow conclusions to be drawn about effectiveness. One of the limitations of this study is that the case studies were not produced for research purposes and therefore lack details and specificity. This is a common challenge in documentary analysis [50] and limits the conclusions that can be drawn from this type of evidence.

There are limitations with the sample. The synthesis mainly included local authorities who were part of the government COVID community champions scheme, although some case studies reported on the adaptation of existing pre-pandemic programmes and others on programme development prior to receiving funding. Community champion schemes were developed by local authorities who did not receive funding during this period; however, we were not aware of any other case study collections at the time of the study. Drawing case studies from a wider sample across a longer time frame (including new programmes developed in response to the 2nd funding round [25]), would have given a better understanding of application at different stages of the pandemic, but we were limited by what was published. Gathering new case studies with different formats, for example focusing on the perspectives of champions, would provide a broader picture of implementation but was beyond the scope of this study. A further limitation is that community champion case studies published on the LGA COVID Learning Exchange collection represent a self-selected sample. The call to submit case studies was open to all local authorities and will reflect those areas that had the time, capacity and capability to respond to that call. While OHID regional staff encouraged submission from local authorities in their area, no selection criteria were applied. Existing relationships with local public health teams, and whether authorities were already communicating their approach through local media, may have facilitated some case studies. However, OHID teams, including the authors (TM, JAS, JS) took an inclusive approach based on the value of disseminating multiple and varied examples of learning. The whole research team then made the decision to include all published case studies of COVID community champions in the later synthesis.

There is potential for bias as case studies may be more likely to be developed when there is a positive story to tell and also more positive aspects maybe emphasised when narratives are being placed in the public domain [51]. In this set of case studies, there was little discussion of what had not worked, conversely there was extensive reflection on emergent learning during the pandemic. Accounts could have been richer in places drawing on alternative perspectives; however, the context of the pandemic and the pressures on staff cannot be understated [52]. The sample potentially represents some of the ‘best available’ evidence on the realities of implementation during a very busy and challenging period for public health [53], when interviews and other data gathering processes might have distracted from front-line delivery. There are wider implications for developing practice-based case studies in public health. Practitioners should be encouraged to provide detailed accounts of learning, which include reflections on what does not work and how implementation evolves from initial plans in response to barriers and changing needs.

Practice-based case studies typically reflect practitioner perspectives on implementation; however, there is potential to include community perspectives as part of the case study [29]. The community champion case studies were generally written by local government staff, including public health practitioners, and in some cases by the VCS organisations involved in programme delivery [eg c2, c12, c13]. This is a major limitation as the experiences of communities, particularly those volunteering as community champions, are not reflected directly, although some case studies reported on results of their community insight work [eg. c1, c8,c13]. In developing public health case studies or practice examples, practitioners should consider the inclusion of community insights. Despite these limitations, we consider that the case studies in this synthesis are valuable data sources around community engagement in an emergency. More research is needed to understand the perspectives of community champions and of communities to complement these professional perspectives.

Overall, the limitations of the data sources limit the conclusions that can be drawn, with the primary risk of bias that themes are drawn from well-established programmes with broadly positive narratives and professional insights dominate. Conversely the case studies analysed in this paper provided rich discussion of learning, identifying both facilitators and constraints and offer important insights into the realities of implementation during this challenging period.

This was a collaborative study initiated through OHID as part of a collaborative research agreement, and then designed and carried out by researchers at Leeds Beckett University. Three of the authors were involved in the initial collation of case studies as part of their OHID roles (TM, JAS, JS) as part of disseminating evidence on community-centred public health. While close collaboration between practitioners and academics can improve knowledge translation and the relevance of research, it meant that we were sensitised to some of the themes and our prior knowledge may have influenced interpretations. To ensure authenticity of the final results in relation to the data, the academic team included independent researchers who had not been involved in the original collation of case studies (JW, AB, AP). Analysis was conducted using recognised qualitative techniques with opportunities for cross checking and validation of results built in at each stage.

Almost all the case studies were written in 2021 and inevitably reflect learning at the time point when completed. From 2020 onwards, pandemic risks, strategies and guidance were constantly changing. Further retrospective case study research is needed to understand the evolution of this approach into the period of pandemic recovery. This paper shows the potential benefits of synthesising learning from practice where time is critical, and the intervention is non-standardised. There is potential for wider application of case study synthesis to other contexts in public health, including identifying learning in other emergencies. Overall, practice-based accounts of public health interventions at community-level form an important resource for later research studies. More primary research is now needed, with well-designed intervention studies to assess processes and outcomes.

## Conclusion

Community champion approaches were widely used by local authorities in England during the COVID-19 pandemic as a means of tackling inequalities and improving connections with disadvantaged communities. This synthesis of practice-based case studies confirms that the community champion model was adaptable when developing local community-centred responses to reduce inequalities and improve access to vaccines. Flexibility in implementation was underpinned by core programme features around recruitment, training, inclusivity, communication and community leadership. From the perspective of public health practice, the primary facilitating factor was seen to be closer relationships between communities and public authorities based on mutual respect and trust. Lessons from this study on volunteer mobilisation and how to engage at-risk populations have wider relevance for public health communication and outreach in outbreak management and more generally for longer term health improvement.

Notwithstanding the limitations of practice-based evidence in public health, this paper shows the potential for synthesising narratives on the practicalities of community engagement in the pandemic. Public health practitioners should be encouraged to develop and disseminate case studies that report emergent learning of what did and did not work because this is valuable and timely information. Where possible community perspectives should be included. Going forward, more research on community champion programmes is needed to capture long term impacts on individual and community health. This should include in-depth research to understand the experiences of community champions and community members during and following the pandemic.

### Supplementary Information

**Additional file 1. **List of data sources for COVID community champion practice-based case study synthesis.

## Data Availability

All the case studies are publicly available on the LGA Public Health Learning Exchange—https://khub.net/web/public-health-learning-exchange.

## Abbreviations

VCS: Voluntary and Community Sector
LGA: Local Government Association
PHE: Public Health England
OHID: Office for Health Improvement and Disparities

## References

[1] World Health Organization Regional Office for Europe (2020). Pandemic fatigue: Reinvigorating the public to prevent COVID-19.

[2] Yardley L, Amlôt R, Rice C, Robin R, Michie S. BMJ Opinion. BMJ. 2020. Available from: https://blogs.bmj.com/bmj/2020/03/17/how-can-we-involve-communities-in-managing-the-covid-19-pandemic/. Cited 2020 4th May.

[3] Questa K, Das M, King R, Everitt M, Rassi C, Cartwright C (2020). Community engagement interventions for communicable disease control in low- and lower- middle-income countries: evidence from a review of systematic reviews. Int J Equity Health.

[4] Schulz AJ, Mehdipanah R, Chatters LM, Reyes AG, Neblett EW, Israel BA (2020). Moving health education and behavior upstream: lessons from COVID-19 for addressing structural drivers of health inequities. Health Educ Behav.

[5] Quinn KG (2020). Applying the popular opinion leader intervention for HIV to COVID-19. AIDS Behav.

[6] Todd A, Bambra C (2021). Learning from past mistakes? The COVID-19 vaccine and the inverse equity hypothesis. Eur J Public Health.

[7] Brunton G, Thomas J, O’Mara-Eves A, Jamal F, Oliver S, Kavanagh J (2017). Narratives of community engagement: a systematic review-derived conceptual framework for public health interventions. BMC Public Health.

[8] Laverack G, Manoncourt E (2016). Key experiences of community engagement and social mobilization in the Ebola response. Glob Health Promot.

[9] Bedson J, Jalloh MF, Pedi D, Bah S, Owen K, Oniba A (2020). Community engagement in outbreak response: lessons from the 2014–2016 Ebola outbreak in Sierra Leone. BMJ Glob Health.

[10] Whittaker J, McLennan B, Handmer J (2015). A review of informal volunteerism in emergencies and disasters: Definition, opportunities and challenges. IJDRR.

[11] Dolan P, Krekel C, Shreedhar G, Lee H, Marshall C, Smith A (2021). Happy to help: The welfare effects of a nationwide micro-volunteering programme. Discussion Paper No.1772.

[12] Gilmore B, Ndejjo R, Tchetchia A, de Claro V, Mago E, Lopes C (2020). Community engagement for COVID-19 prevention and control: a rapid evidence synthesis. BMJ Glob Health.

[13] Public Health England (2015). NHS England.

[14] Royal Society for Public Health (2014). Tackling health inequalities: the case for investment in the wider public health workforce.

[15] National Institute for Health and Care Excellence (2016). Community engagement: improving health and wellbeing and reducing health inequalities.

[16] South J, Meah A, Bagnall A-M, Jones R (2013). Dimensions of lay health worker programmes: results of a scoping study and production of a descriptive framework. Glob Health Promot.

[17] Public Health England (2021). Community champions. A rapid scoping review of community champion approaches for the pandemic response and recovery.

[18] Shelton RC, Dunston SK, Leoce N, Jandorf L, Thompson HS, Erwin DO (2017). Advancing understanding of the characteristics and capacity of African American women who serve as lay health advisors in community-based settings. Health Educ Behav.

[19] Rhodes SD, Foley KL, Zometa CS, Bloom FR (2007). Lay health advisor interventions among hispanics/latinos a qualitative systematic review. Am J Prev Med.

[20] Community Champions to give COVID-19 vaccine advice and boost take up [Press release]. 25^th^ January 2021, gov.uk. Available from: https://www.gov.uk/government/news/community-champions-to-give-covid-19-vaccine-advice-and-boost-take-up.

[21] Public Health England (2020). Disparities in the risk and outcomes of COVID-19.

[22] Public Health England (2020). Beyond the data: Understanding the impact of COVID-19 on BAME groups.

[23] Scientific Advisory Group for Emergencies S-B. The role of Community Champion networks to increase engagement in the context of COVID-19: Evidence and best practice. 2020. Available from: https://www.gov.uk/government/publications/role-of-community-champions-networks-to-increase-engagement-in-context-of-covid-19-evidence-and-best-practice-22-october-2020.

[24] Race Disparity Unit, Cabinet Office. Third quarterly report on progress to address COVID-19 health inequalities, gov.uk. 2021. Available from: https://www.gov.uk/government/publications/third-quarterly-report-on-progress-to-address-covid-19-health-inequalities/third-quarterly-report-on-progress-to-address-covid-19-health-inequalities.

[25] National effort to vaccinate vulnerable communities receives funding boost. [Press release], 13th January 2022, gov.uk. Available from: https://www.gov.uk/government/news/national-effort-to-vaccinate-vulnerable-communities-receives-funding-boost.

[26] New era of public health to tackle inequalities and level up the UK [press release]. 1st October, 2021, gov.uk. Available from: https://www.gov.uk/government/news/new-era-of-public-health-to-tackle-inequalities-and-level-up-the-uk.

[27] Local Government Association. Public Health Learning Exchange, undated. Available from: https://khub.net/web/public-health-learning-exchange.

[28] Zwald M, Jernigan J, Payne G, Farris R (2013). Developing stories from the field to highlight policy, systems, and environmental approaches in obesity prevention. Prev Chronic Dis.

[29] South J, Mapplethorpe T, Gledhill R, Marsh W, Stansfield J, Evans S, et al. Learning from public health practice: the development of a library of community-centered practice examples. J Public Health. 2022;45(2):414–22. 10.1093/pubmed/fdac065 PMC10273362 35774035

[30] Shankardass K, Renahy E, Muntaner C, O’Campo P (2014). Strengthening the implementation of health in all policies: a methodology for realist explanatory case studies. Health Policy Plan.

[31] Morestin F, Gauvin F, Hogue M, Benoit F (2010). Method for synthesizing knowledge about public policies.

[32] Simpson S, Kelly MP, Morgan M (2013). Defining principles for good practice: using case studies to inform health systems action on inequalities. Eval Program Plann.

[33] Van Wynsberghe R, Khan S (2007). Redefining case study. Int J Qual Methods.

[34] Lee KS, Chavis DM (2012). Cross-case methodology: Bringing rigour to community and systems change research and evaluation. J Community Appl Soc Psychol.

[35] Simpson S, Kelly MP, Morgan A (2013). Defining principles for good practice: Using case studies to inform health systems action on health inequalities. Eval Program Plann.

[36] Khan S, Van Wynsberghe R. Cultivating the under-mined: Cross-case analysis as knowledge mobilization. Forum Qual Soc Res. 2008;9(1):Art 43. Available at https://www.qualitative-research.net/index.php/fqs/article/view/334/730.

[37] Hardoon D, South J, Southby K, Freeman C, Bagnall A, Pennington A (2021). A guide to synthesising case studies.

[38] Taylor-Collins E, Havers R, Durrant H, Passey A, Bagnall AM, South J (2021). Volunteering and wellbeing in the pandemic. Part I: Learning from practice.

[39] UK Health Security Agency Knowledge & Library Services. Practice Examples undated. Available from: https://ukhsalibrary.koha-ptfs.co.uk/practice-examples/.

[40] Gale NK, Heath G, Cameron E, Rashid S, Redwood S (2013). Using the framework method for the analysis of qualitative data in multi-disciplinary health research. BMC Med Res Methodol.

[41] Shepherd JL, O’Caña F (2013). Committed to the community: the Atlas HIV Prevention Program. Health Promot Pract.

[42] Rice RE, Wu Z, Li L, Detels R, Rotheram-Borus MJ (2012). Reducing STD/HIV stigmatizing attitudes through community popular opinion leaders in Chinese markets. Hum Commun Res.

[43] Libyan Red Crescent Society. Volunteer in every street - webinar. Geneva: WHO Infodemic Management and Global Collective Service; 2020.

[44] Holt M, Ruiz-Aguilera E, Ngeno G, Bronner Y. Healing Baltimore: Creating Support for VALUE (Vaccine Access & Acceptance, Lives in Unity, Education & Engagement) Baltimore Ambassadors as They Serve During the COVID-19 Pandemic. Health Promot Pract. 2023:15248399231166714. 10.1177/15248399231166714. 10.1177/15248399231166714 PMC10113907 37073499

[45] Nunes NRDA (2021). The power that comes from within: female leaders of Rio de Janeiro’s favelas in times of pandemic. Glob Health Promot.

[46] Morgan GT, Poland B, Jackson SF, Gloger A, Luca S, Lach N (2021). A connected community response to COVID-19 in Toronto. Glob Health Promot.

[47] South J, Bagnall A-M, Stansfield J, Southby K, Mehta P (2019). An evidence-based framework on communitycentred approaches for health: England UK. Health Promot Int.

[48] Mankell A, Abdelzadeh A. The role of community trust for compliance with the Swedish COVID-19 immunisation programme. Scand J Public Health. 2023;51(5):704–10. 10.1177/14034948221145780 PMC9829506 36609189

[49] Bollyky TJ, Hulland EN, Barber RM, Collins JK, Kiernan S, Moses M (2022). Pandemic preparedness and COVID-19: an exploratory analysis of infection and fatality rates, and contextual factors associated with preparedness in 177 countries, from Jan 1, 2020, to Sept 30, 2021. Lancet.

[50] Bowen GA (2009). Document analysis as a qualitative research method. Qual Res J.

[51] Centers for Disease Control and Prevention (2008). How to develop a success story.

[52] Shand W, Jarvis S (2021). Responding to COVID-19 in the Liverpool City Region. The impact of COVID-19 on methods and approaches to community-based participatory research.

[53] Lancaster K, Rhodes T, Rosengarten M (2020). Making evidence and policy in public health emergencies: lessons from COVID-19 for adaptive evidence-making and intervention. Evid Policy.

