# Supplementary material for: A qualitative synthesis of practice-based learning from case studies on COVID community champion programmes in England, UK
Source: BMC Public Health. 2024 Jan 2;24:7. doi: 10.1186/s12889-023-17470-1 (PMC10759547; doi:10.1186/s12889-023-17470-1)
Supplement: Supplementary file 1 — Additional file 1. List of data sources for COVID community champion practice-based case study synthesis. [file 12889_2023_17470_MOESM1_ESM.doc]

**Additional File 1:** **List of data sources for COVID community champion practice-based case study synthesis**

| **Code** | **Local Authority area** | **Date published by LGA** | **Date updated by LGA** | **Published in ‘Community Champions (DLUHC^[[1]](#footnote-1)^) resources’** | **Title** | **URL** | **Programme summary (from within-case analysis)** | **Date 1st accessed** | **Date Last accessed** |
| --- | --- | --- | --- | --- | --- | --- | --- | --- | --- |
| C1 | Arun | 10/12/2021 | 17/12/2021 | YES | Arun District Council - Community Champions | <https://khub.net/web/public-health-learning-exchange/resources/-/ddl_display/ddl/578500182/723707339/maximized> | Community champion programme was a major component of partnership work to increase the reach and effectiveness of COVID communications. Delivery through VCS organisations who recruited, trained and supported champions. | 28/04/2022 | 20/06/2023 |
| C2 | Bradford | 15/09/2021 | 08/10/2021 | YES | Covid-19 Community Champions - Bradford & District | <https://khub.net/web/public-health-learning-exchange/resources/-/ddl_display/ddl/526607348/723707339/maximized> | Creation of network of community partners (grassroots organisations and faith groups), coordinated by Race Equality Network. Lead champions recruited other champions and facilitated 2-way information flows with communities. | 28/04/2022 | 20/06/2023 |
| C3 | Calderdale | 17/01/2022 | 28/01/2022 | YES | Calderdale COVID-19 Champions | <https://khub.net/web/public-health-learning-exchange/resources/-/ddl_display/ddl/593983930/723707339/maximized> | Community-initiated programme, later rolled out across district. Focused on creation of ‘network of informed and aware citizens’ who raised awareness and understanding of vaccination and managing COVID risk. | 28/04/2022 | 20/06/2023 |
| C4 | Doncaster | 31/10/2022 | 04/01/2023 | YES | Covid Community Champions- Doncaster City | <https://khub.net/web/public-health-learning-exchange/resources/-/ddl_display/ddl/787314425/723707339/maximized> | Pre-existing community connector programme adapted to the pandemic response. Community connectors were employed by VCS organisations to connect with communities and help design culturally sensitive services. Approach based on asset-based principles using appreciative inquiry as basis for community conversations. | 23/09/2022 | 20/06/2023 |
| C5 | Gateshead | 13/12/2021 | Not updated | YES | Gateshead Community Champions Programme | <https://khub.net/web/public-health-learning-exchange/resources/-/ddl_display/ddl/571677182/723707339/maximized> | Population-wide approach focused on capacity building in workplace and community settings. Pre-existing MECC scheme extended to other vulnerable groups and settings. Training supported champions to cascade information to communities. | 28/04/2022 | 20/06/2023 |
| C6 | Hyndburn | 05/08/2021 | 08/10/2021 | YES | COVID-19 Community Champions – Hyndburn | <https://khub.net/web/public-health-learning-exchange/resources/-/ddl_display/ddl/511475785/723707339/maximized> | Strengthening local infrastructure and collaboration with VCS organisations to improve outreach, communication and volunteering. Champions (people or organisations) worked within community action network. | 28/04/2022 | 20/06/2023 |
| C7 | Bi-Borough (Royal Borough of Kensington & Chelsea/City of Westminster) | 10/08/2021 | 16/08/2021 | YES | Bi-Borough (Royal Borough of Kensington & Chelsea/City of Westminster) Community Champions | <https://khub.net/web/public-health-learning-exchange/resources/-/ddl_display/ddl/511475887/723707339/maximized> | Pre-existing health champions programme based on neighbourhood work, community and personal development. Additional COVID health champions role to support pandemic response. Programme gathered community insight to shape services and address inequalities. | 28/04/2022 | 20/06/2023 |
| C8 | Kirklees | 31/10/2022 | 04/01/2023 | YES | Community Champions Kirklees West Yorkshire | <https://khub.net/web/public-health-learning-exchange/resources/-/ddl_display/ddl/787314092/723707339/maximized> | Community champion programme focus on improving communication and vaccine uptake. Grants given to VCS organisations and groups to reach target communities, recruit champions and build capacity for engagement activities. A learning & support network facilitated communication and sharing community insight. | 23/09/2022 | 20/06/2023 |
| C9 | Manchester | 14/06/2021 | 12/10/2021 | YES | COVID Health Equity Manchester (CHEM) | <https://khub.net/web/public-health-learning-exchange/resources/-/ddl_display/ddl/511457438/723707339/maximized> | Establishment of a health equity collaboration to improve messaging and communication to at-risk populations. Supported 2-way information flow with communities. Range of volunteer roles including community influencers using mass media and COVID chat volunteers. | 28/04/2022 | 20/06/2023 |
| C10 | Middlesbrough | 17/10/2021 | 18/10/2021 | YES | COVID-19 Community Champions Network - Middlesbrough Borough Council | <https://khub.net/web/public-health-learning-exchange/resources/-/ddl_display/ddl/543405302/723707339/maximized> | COVID champions network was key mechanism supporting 2-way information flows with communities. Grew from network of funded VCS organisations to include staff, residents and community leaders. Public health facilitated network and provided grants. | 28/04/2022 | 20/06/2023 |
| C11 | Rochdale | 04/10/2021 | 08/102021 | YES | COVID-19 Community Champions - Rochdale Borough Council | <https://khub.net/web/public-health-learning-exchange/resources/-/ddl_display/ddl/538535623/723707339/maximized> | Comprehensive community champions programme built on pre-existing whole system communities strategy. Communication and gathering community insights occurred through local networks and COVID community engagement groups. Champions discussed wider health determinants as well as pandemic issues. | 28/04/2022 | 20/06/2023 |
| C12 | Slough | 19/08/2021 | 23/08/2021 | YES | One Slough Wellbeing Friends | <https://khub.net/web/public-health-learning-exchange/resources/-/ddl_display/ddl/515459875/723707339/maximized> | Programme focused on community support within pandemic. Volunteering service based on ‘wellbeing friends’ offering 1:1 telephone support to increase wellbeing and reduce social isolation of vulnerable adults. Also a wider champions network to improve messaging in communities. | 24/04/2022 | 20/06/2023 |
| C13 | Harrow | 10/12/2021 | 17/12/2021 | YES | Voluntary Action Harrow - Community Champions | <https://khub.net/web/public-health-learning-exchange/resources/-/ddl_display/ddl/578548175/723707339/maximized> | Four case studies developed by VCS organisations; 3 representing specific ethnic minority populations and 1 working with young people from diverse ethnic minority communities.  VCS groups received grants, mobilised staff, volunteers and community leaders and led design and delivery of engagement activities through community networks. | 24/04/2022 | 20/06/2023 |
| C14 | Birmingham | 03/02/2021 | 23/08/2021 | NO | Birmingham City Council: Training community champions to address vaccine concerns | <https://www.local.gov.uk/birmingham-city-council-training-community-champions-address-vaccine-concerns> | Programme developed early in pandemic initially to support COVID messaging, later used for vaccine roll out. Champions as resource in public health system because they tackled barriers and built trust. | 28/04/2022 | 20/06/2023 |
| C15 | Leeds | 24/02/2022 | Not updated | NO | Leeds: COVID-19 Community Champions Programme | <https://www.local.gov.uk/case-studies/leeds-covid-19-community-champions-programme-january-march-2022> | Programme built a network of champions and local grassroots organisations. Mix of supporting local health services and community-led outreach activities. Some activities supported by small grants. Emphasis on community empowerment and working with trusted messengers with cultural competence and language skills. | 20/09/2022 | 20/06/2023 |
| C16 | Peterborough | 03/02/2021 | 23/08/2021 | NO | Cambridgeshire and Peterborough: working with the whole community | <https://www.local.gov.uk/cambridgeshire-and-peterborough-working-whole-community> | Community champions integral to partnership approach across various levels of local government. Emphasis on champions as trusted messengers who communicated messages, tackled disinformation and translated. | 28/04/2022 | 20/06/2023 |

1. DLUHC – Department for Levelling Up, Housing and Communities [↑](#footnote-ref-1)
